# Preparation and Characterization of Softwood Kraft Lignin Copolymers as a Paper Strength Additive

**DOI:** 10.3390/polym10070743

**Published:** 2018-07-05

**Authors:** Zhongming Liu, Dingding Xu, Lei Xu, Fangong Kong, Shoujuan Wang, Guihua Yang

**Affiliations:** 1 State Key Laboratory of Biobased Material and Green Papermaking, Key Laboratory of Pulp & Paper Science and Technology of Shandong Province/Ministry of Education, Qilu University of Technology (Shandong Academy of Sciences), Jinan 250353, China; liuzhongming126@126.com (Z.L.); albertine_dd@163.com (D.X.); ygh2626@126.com (G.Y.); 2 Xuancheng Product Quality Supervision and Inspection Institute, Xuancheng 242000, China; sdd3166@126.com

**Keywords:** softwood kraft lignin (SKL), SKL copolymers, paper additive, physical properties

## Abstract

Softwood kraft lignin is a renewable type of woody material that can be converted to value-added products, for example, as a paper strength additive in the paper industry. In this study, the monomers of methacryloxyethyltrimethyl ammonium chloride (DMC), acrylic acid (AA), and acrylamide (AM) were grafted on softwood kraft lignin (SKL) to prepare three different SKL copolymers. Fourier-transform infrared, proton nuclear magnetic resonance, charge density, elemental, and molecular weight analyses confirmed that the monomers were successfully grafted onto SKL. The grafting rates of SKL-DMC, SKL-AA, and SKL-AM copolymers were 80.35%, 82.70%, and 79.48%, respectively. The application of SKL copolymers as a paper additive for enhancing paper physical properties was studied. The results indicated that at a 2 wt % dosage of SKL copolymers, the increase in the physical properties of paper is maximum.

## 1. Introduction

Lignin is the second-most abundant natural aromatic (phenolic) polymer in nature after cellulose and is used in several industrial operations with significant sustainability [1,2]. The worldwide generation of industrial lignin has reached several hundred million tons annually [3], and lignin has been recognized as a potential raw material for preparing value-added products, such as phenols, sinnapyl alcohol [4], vanillin [5], lignin-based materials [6], and other biomass-based products [7,8]. In order to fully utilize this low-cost resource, chemical modification is considered one of the effective techniques for altering the properties of lignin and promoting the value-added nature of the products [9]. Previously, modification reactions of lignin have been reported in the literature, such as oxidation [10], carboxymethylation [8], and copolymerization [11,12,13].

Copolymerization, which is one of the most effective chemical modification techniques, was applied in the preparation of water-soluble kraft lignin–acrylic acid copolymer [14]. Mohamad and co-workers produced a lignin graft copolymer as a mud thinner for deep-well drilling operations [15]. Nguyen and co-workers reported the preparation of lignin-based thermoplastic copolyester for eco-friendly polymers using kraft lignin [16]. In addition, cationic Xylan copolymer was used as a flocculant for removing dyes from the wastewater in the textile industry [17]. Wang and co-workers also developed a soda lignin–acrylamide (AM) copolymer as a paper dry-strength additive [18]. However, the use of lignin and its derivatives as strength additives in the paper-making industry as a pulp-strengthening agent is still quite low [18]. The chemi-mechanical pulp varies, as raw material of paper usually has low strength properties, especially tensile, burst, and tear strength. This low strength of chemi-mechanical pulp has hampered its utilization in furnish preparation in the paper-making industry. It is necessary to add a strengthening agent in the pulp to give the final desired paper product good strength properties. It is known that different monomers contain different functional groups which could give different properties to the final polymers. Here, methacryloxyethyltrimethyl ammonium chloride (DMC), acrylic acid (AA), and acrylamide (AM) were chosen as functional monomers, based on previous literature, to polymerize with softwood kraft lignin. Of course, there may be other monomers that could be used, but they would produce different results for paper strength improvement.

In this paper, softwood kraft lignin copolymers were prepared through a polymerization reaction. The prepared softwood kraft lignin copolymers were used as a strengthening agent for paper-making. The main novelties of this work are the following: (1) the preparation of softwood kraft lignin copolymers and copolymer characterization using various analytical techniques, and (2) the analysis and comparison of strengthening performances of three different softwood kraft lignin copolymers in terms of their physical properties as additives in paper.

## 2. Materials and Methods

### 2.1. Materials

Softwood kraft lignin (SKL) was produced from black liquor from kraft pulping through an acid precipitation process [11]. Methacryloxyethyltrimethyl ammonium chloride (DMC), acrylic acid (AA), and acrylamide (AM) were all purchased from Sigma-Aldrich Co. (Shanghai, China). Potassium persulfate was obtained from Sigma-Aldrich and used as initiator reagent without further purification. Polydiallyldimethylammonium chloride (PDADMAC) and potassium polyvinyl sulfate (PVSK) were purchased from Sigma-Aldrich and diluted to 0.001 M prior to use. The alkaline peroxide mechanical pulp (APMP) used in this experiment was obtained from Shandong Sun Paper Industry (Yanzhou, Chian). The APMP pulp properties including chemical components were analyzed according to TAPPI standards (TAPPI 1996) and are listed in Table 1.

### 2.2. Preparation of SKL Copolymer

In this set of experiments, 2 g of SKL was mixed with 40 mL of deionized water in a 250 mL three-neck glass flask under stirring at 400 rpm. A 0.1 mol/L NaOH solution was then gradually added into the solution to adjust the initial pH to 11. After 30 min of stirring, the pH of the solution was adjusted to 4 using 0.1 mol/L sulfuric acid at 70 °C. The flask was kept in a water bath, and the solution was deoxygenated using atmosphere for 30 min. Then, 0.03 g of potassium persulfate was added to the solution as an initiator under stirring at 400 rpm. After 10 min, SKL/monomer molar ratios of one-third were added drop-wise to the solution and stirred at 400 rpm for 3 h at 75 °C. After completion of the reaction, the solution was cooled to room temperature, and the solution was mixed with 80 vol. % ethanol/water in order to precipitate SKL copolymer from the rest of the reaction medium. The precipitate was then washed with 80 vol. % ethanol twice and dried in an oven at 105 °C to generate the purified SKL copolymers (SKL-DMC, SKL-AA, and SKL-AM).

### 2.3. Analytical Methods

#### 2.3.1. FTIR Analysis

Fourier-transform infrared (FTIR) analysis was conducted on the SKL and SKL copolymer samples using a FTIR spectrophotometer (Bruker VERTEX70, Rheinstetten, Germany). In this measurement, 0.01 g samples of SKL and SKL copolymers were used. Each spectrum was recorded with 32 scans in transmittance mode with a resolution of 0.5 cm^−1^ within the range of 400–4000 cm^−1^.

#### 2.3.2. Proton Nuclear Magnetic Resonance Analysis

The proton nuclear magnetic resonance (^1^H-NMR) analyses of SKL and SKL copolymer samples were performed using a NMR spectrometer (Bruker AVANCE II 400 MHz, Rheinstetten, Germany) with an acquisition time of 0.011 s at room temperature. ^1^H-NMR analysis was carried out by dissolving approximately 10 mg of the samples in 0.5 mL of D_2_O, and the spectrum was directly recorded over 32 scans.

#### 2.3.3. Thermal Analysis

Thermal analyses of SKL and SKL copolymer samples were performed using a thermogravimetric analyzer (TGA Q50, New Castle, DE, USA). Samples of 3–10 mg were used in this analysis from room temperature to 600 °C at a rate of 10 °C/min under an N environment.

#### 2.3.4. Molecular Weight Analysis

Approximately 5 mg of dried SKL and SKL copolymer samples were dissolved in 0.1 mol/L NaNO_3_ by stirring at 500 rpm for 36 h at 35 °C, and then the solutions were filtered with a 0.2 µm nylon filter. The filtered solutions were used for the molecular weight analysis of the samples, which was carried out using gel permeation chromatography (GPC) with a Heleos-II GP chromatograph (Wyatt Technology, Santa Barbara, CA, USA) with a multi-angle laser light-scattering detector. The columns of PolyAnalytic PAA 206 and PAA 203 were set up at 35 °C, and 0.1 mol/L NaNO_3_ solution was used as the solvent and eluent. The flow rate was set at 0.50 mL/min, while poly(ethylene oxide)s were used as standard samples for calibration of this aqueous system.

#### 2.3.5. Elemental Analysis

Elemental analyses of SKL and SKL copolymer samples were carried out with an elemental analyzer (Vario EL III, Elementar Analysen Systeme, Hanau, Germany). Approximately 2–5 mg samples of SKL and SKL copolymer were combusted while the temperature rose to 1150 °C.

#### 2.3.6. Charge Density Analysis

In this set of experiments, SKL and SKL copolymer samples were initially dried in a 105 °C oven overnight to remove moisture. A 0.02 g sample was then dissolved in 100 mL of deionized water and incubated for 1 h at 30 °C in a water bath shaker at 150 rpm. After incubation, the charge densities of the SKL and SKL copolymer samples were determined with PDADMAC and PVSK standard solution (0.001 mol/L) using a particle charge detector (Mutek, PCD 04, Herrsching, Germany).

#### 2.3.7. Grafting Ratio

The grafting ratio of SKL-DMC was identified using Equation (1), which was described in the work on the cationic Xylan-METAC copolymer [17,19]:

The aqueous potentiometric titration method was used to measure the carboxylate group content of SKL-AA copolymer samples using an automatic potentiometer (Metrohm, 905 Titrado, Herisau, Switzerland). In this set of experiments, a 1 g sample of SKL-AA copolymer was added to 100 mL of distilled water, and the pH of the solution was adjusted to 10.5. The solution was then titrated with a cationic surfactant, TEGOtrant A100, to measure the number of carboxylate groups. The grafting ratio was calculated using Equation (2) [20]:

(1)  grafting ratio=(C×Mw)/(100−C×Mw)×100

(2)  C=N/n

where *N* is the nitrogen content of the samples (wt %), and *Mw* (DMC) is the molecular weight of DMC (207.7 g/mol). *Mw* (AA) is the molecular weight of AA (72 g/mol). *Mw* (AM) is the molecular weight of AM (71 g/mol). *C* is the total carboxylate group content (mol/g), and *n* is the relative molar mass of *N* (14 g/mol).

#### 2.3.8. Performance Assessments of SKL Copolymer as a Strengthening Agent in Paper-Making

The performance of the SKL copolymer used as strengthening additives was evaluated using APMP, a chemi-mechanical pulp that is viable as a raw material for almost all paper grades. In this experiment, the SKL copolymers were first dissolved in water by stirring for 1 h at 40 °C. Then, different amounts of SKL copolymers, e.g., 1.0–4.0% (wt %, on dried pulp weight), were added into a 1% pulp slurry. The pulp slurry containing SKL copolymer was stirred at 1000 rpm for 5 min before hand sheet formation. Hand sheets were made by pouring the pulp slurry into the bowl of a paper sheet former, followed by draining the slurry through a metallic wire. Each formed hand sheet was dried on a rapid dryer at 97 °C for 7 min. Paper sheets with a grammage of 60 g/m^2^ were used to test tensile index and tear index, and paper sheets with a grammage of 100 g/m^2^ were used to test the burst index and internal bond strength. Before the testing of physical strength properties, the hand sheets were kept in an environment of constant temperature (23 °C) and humidity (50%) for 24 h. Three physical strength properties tests (tensile index, tear index, and burst index) were then separately performed according to the Tappi standard method [18]. All the data presented in this paper represents the average value of three repetitions.

## 3. Results

### 3.1. Preparation of SKL Copolymers

**Figure 1:**
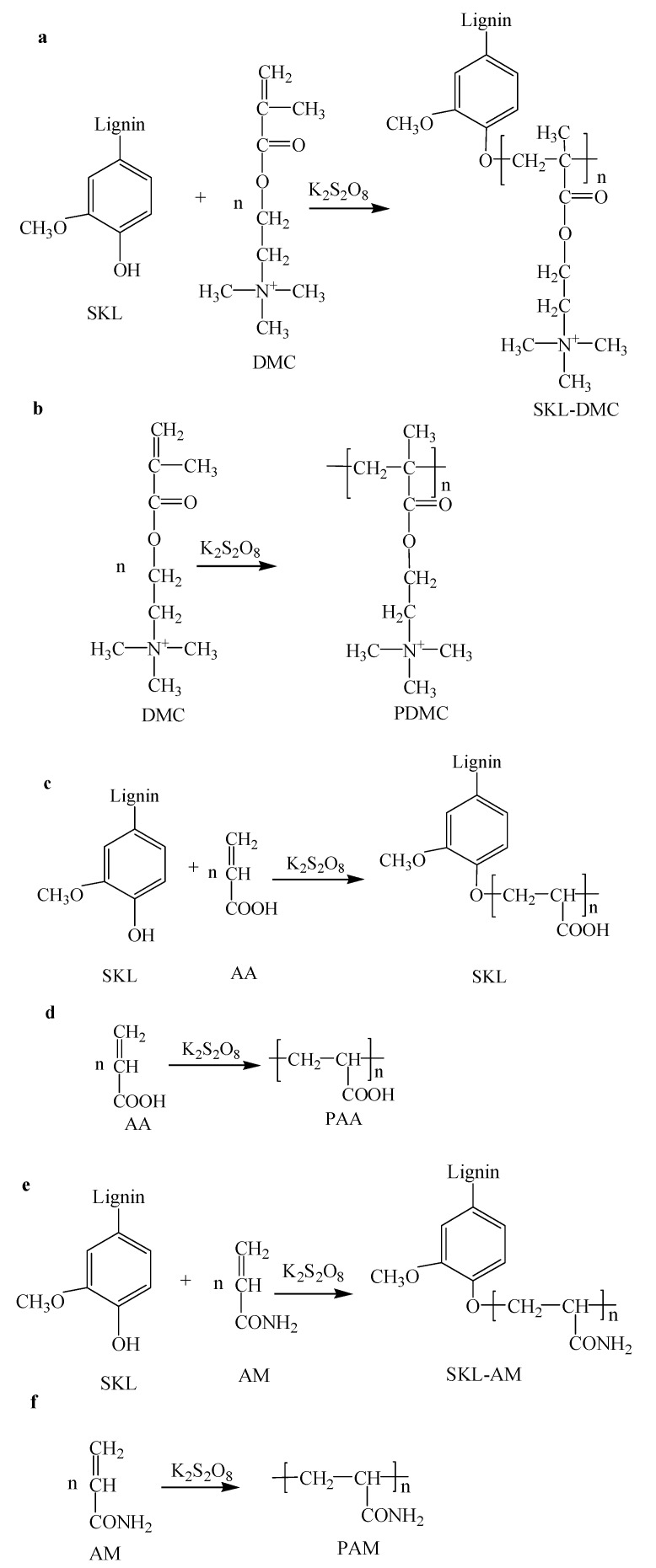
Mechanism of the copolymerization of softwood kraft lignin (SKL) with monomers ((**a**) SKL-DMC, (**b**) PDMC, (**c**) SKL-AA, (**d**) PAA, (**e**) SKL-AM, (**f**) PAM).

The reaction mechanisms of SKL and DMC, AA, and AM are shown in Figure 1. By adding potassium persulfate to the SKL solutions, a lignin free radical was generated at the phenolic hydroxyl group. The alkenyl group of monomers is very active and generates monomer free radicals, which become the acceptor of the free radical of lignin, resulting in chain initiation of the SKL copolymers (Figure 1a: SKL-DMC, Figure 1c: SKL-AA, and Figure 1e: SKL-AM) [14,17]. Furthermore, the monomers can participate in a side reaction to produce homopolymers of Poly(methacryloxyethyltrimethyl ammonium chloride) (PDMC) (Figure 1b), Poly acryl acid (PAA) (Figure 1d), and Polyacrylamide (PAM) (Figure 1f).

### 3.2. FTIR Analysis

**Figure 2:**
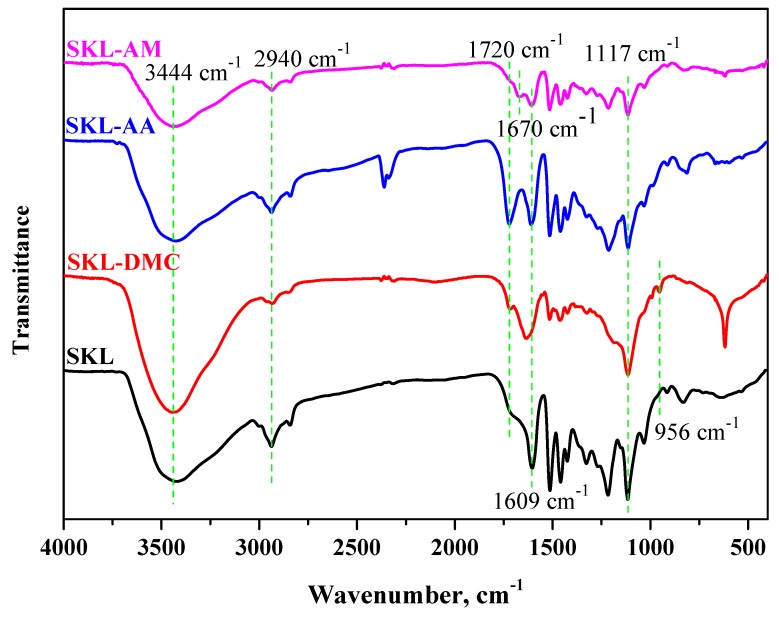
Fourier-transform infrared (FTIR) spectra of SKL and SKL copolymers.

The FTIR spectra of the SKL and SKL copolymers are shown in Figure 2. The absorption peak at 3444 cm^−1^ was attributed to the hydroxyl stretching vibration in aromatic and aliphatic groups in the SKL and SKL copolymers [21]. The three strong absorption peaks at 1609, 1514, and 1456 cm^−1^ were assigned to aromatic skeletal vibrations from SKL and SKL copolymers [22,23], while that at 2928 cm^−1^ was assigned to the C–H stretching vibration [8,24]. However, the SKL-DMC copolymer has two characteristic peaks at 1720 and 956 cm^−1^ due to the C=O stretching vibration and quaternary ammonium group, respectively [17]. The emergence of these two peaks indicates that the DMC monomer was successfully polymerized onto the SKL. Compared with the spectrum of SKL, the spectrum of the SKL-AA copolymer appears to have a new peak at 1720 cm^−1^ due to the stretching vibration of the carboxylate group [25]. The emergence of a new functional group confirms that the SKL-AA copolymer was successfully produced. Compared to the FTIR spectrum of SKL, that of the SKL-AM copolymer appears with two peaks observed at 1670 and 1609 cm^−1^, which were attributed to the carbonyl groups and the C–N stretching vibration of the amide groups, respectively [18]. A new absorption peak was also observed in the spectrum of the SKL-AM copolymer, showing that the SKL was successfully copolymerized with AM.

### 3.3. ^1^H-NMR Analysis

**Figure 3:**
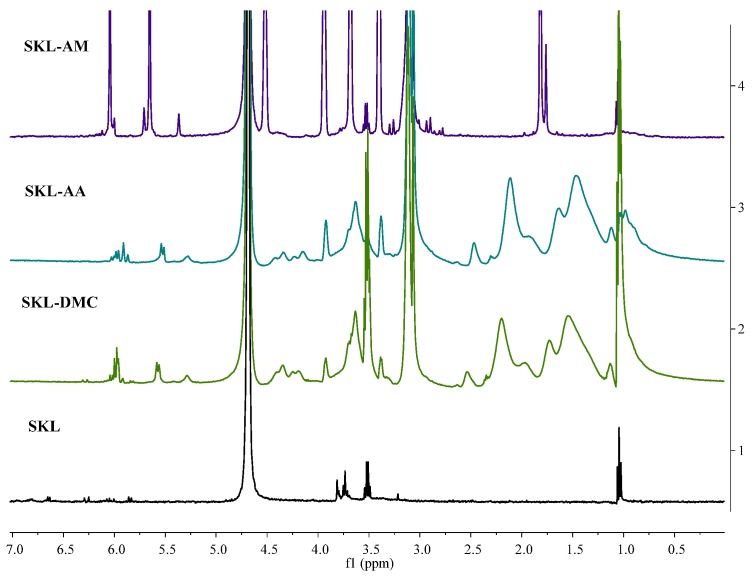
^1^H-NMR spectra of SKL and SKL copolymers.

The ^1^H-NMR spectra of the SKL and SKL copolymers are shown in Figure 3. The peaks at 7.0–6.0 ppm were attributed to aromatic protons, including certain vinyl protons on the carbon atoms adjacent to the aromatic rings. The peaks at 5.9–5.1 ppm were attributed to aliphatic protons, including Hα and Hβ. The peaks at 4.0–3.0 ppm were attributed to protons in methoxyl groups of SKL [26], and the peak at 3.2 ppm was assigned to the methylene protons in the β-β structure from SKL. The peaks at 0.9–1.1 ppm arose from the –CH_3_ protons in the main chain [27]. The peak appearing at 4.5–5.0 ppm was assigned to the protons of the solvent (D_2_O).

Compared with the spectrum of SKL, the signal of the characteristic protons of –N^+^(CH_3_)_3_ is visible at 3.4 ppm, and the peaks in the vicinity of 4.3 and 3.9 ppm originate from the methylene protons in –O–CH_2_–CH_2_– in the SKL-DMC polymer [28]. The peaks appearing at 1.5–2.5 ppm were assigned to the internal reference (TMSP) [29]. The ^1^H-NMR results indicate successful copolymerization of all the functional monomers. By comparing the spectrum of SKL, the peaks appearing at 1.4–2.2 ppm were attributed to C–H and the peak at 2.6 ppm was attributed to the hydroxyl end in the SKL-AA polymer [30]. In addition, a peak at 4.3 ppm was observed in the spectrum of the SKL-AA copolymer, which was assigned to CH_2_–O– connected with a lignin unit structure (Kang, Chen, Wang, and Yang, 2014). New absorption peaks were also observed in the spectrum of the SKL-AA copolymer, showing that the SKL was successfully copolymerized with AA. In the SKL-AM copolymer spectrum, a peak at 4.5 ppm was observed in the spectrum of the SKL-AM copolymer, which was assigned to the protons connecting –CH_2_– to the aromatic structures through ester bonding (–CH_2_–O–C_6_H_5_) [18]. In addition, the peak at 1.8 ppm was observed in the spectrum of the SKL-AM copolymer, which was assigned to the protons connecting the amide groups of the copolymer [31].

### 3.4. Thermogravimetric Analysis

**Figure 4:**
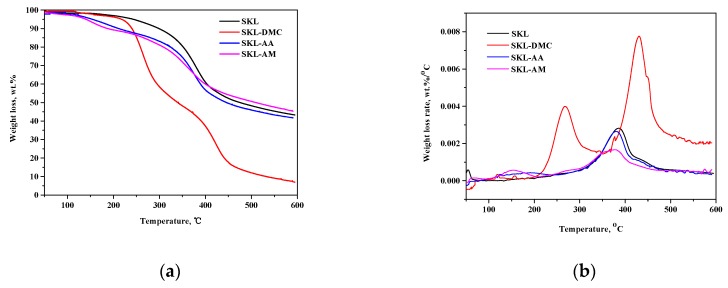
(**a**) Weight loss of SKL and SKL copolymers; (**b**) weight loss rate of SKL and SKL copolymers.

The thermal characteristics of SKL and SKL copolymers are shown in Figure 4. It can be seen that the main degradation temperature range of SKL ranged from 250 °C to 450 °C; however, that of the SKL-DMC sample ranged from 200 °C to 500 °C, and that of the SKL-AA and SKL-AM copolymers ranged from 150 °C to 450 °C. The SKL sample is full of aromatic rings with various branches, and the activity of the chemical bonds in lignin covers an extremely wide range, which led to the degradation of lignin occurring in the entire temperature range from 250 °C to 600 °C [2]. At the end of the analysis, the SKL, SKL-AA, and SKL-AM samples yielded approximately 40 wt % residue char, while SKL-DMC yielded approximately 10 wt % residue char. The above analysis suggests that the chemical modification leads to lower thermal stability of the final products, and this is mainly due to the degradation of the new access groups, as stated in previous works [18,32,33].

### 3.5. Properties of SKL and SKL Copolymers

The properties of the SKL and SKL copolymers are shown in Table 2. It was observed that the charge densities of the SKL-DMC, SKL-AA, and SKL-AM copolymers dramatically increased to +1.425, −6.287, and −3.656 mmol/g, respectively. The grafting ratios of the SKL-DMC, SKL-AA, and SKL-AM copolymers were also shown to be 80.35%, 82.70%, and 79.48%, respectively. The SKL copolymers had a significantly higher weight average molecular weight (*Mw*) and number average molecular weight (*Mn*) compared to those of unmodified lignin. The polydispersity (*Mw/Mn*) of SKL and SKL copolymers show the distribution of molecular weight before and after modification. Furthermore, the N content of lignin–DMC and lignin–AM increased, which was due to the amide groups grafted to SKL. Therefore, based on above analysis, the copolymerization of monomers and SKL successfully altered the elemental components and charge density of SKL.

### 3.6. Application of SKL Copolymers as a Strengthening Agent in Paper Making

**Figure 5:**
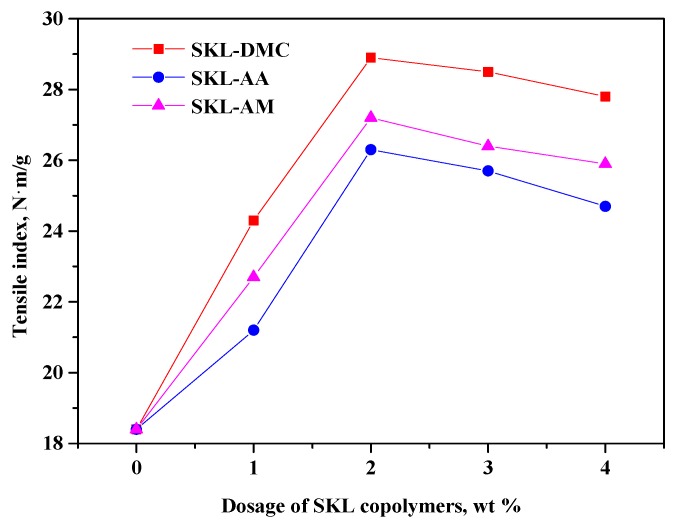
Tensile index of paper sheets with various amounts of three additives added (SKL-DMC, SKL-AA, and SKL-AM).

**Figure 6:**
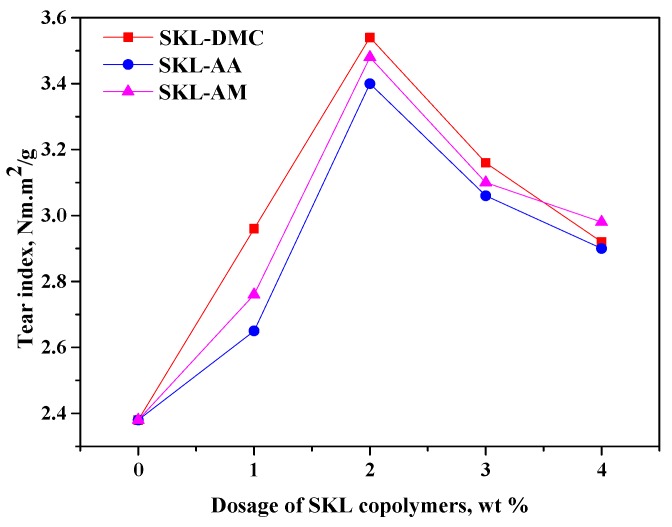
Tear index of paper sheets with various amounts of three additives added (SKL-DMC, SKL-AA, and SKL-AM).

**Figure 7:**
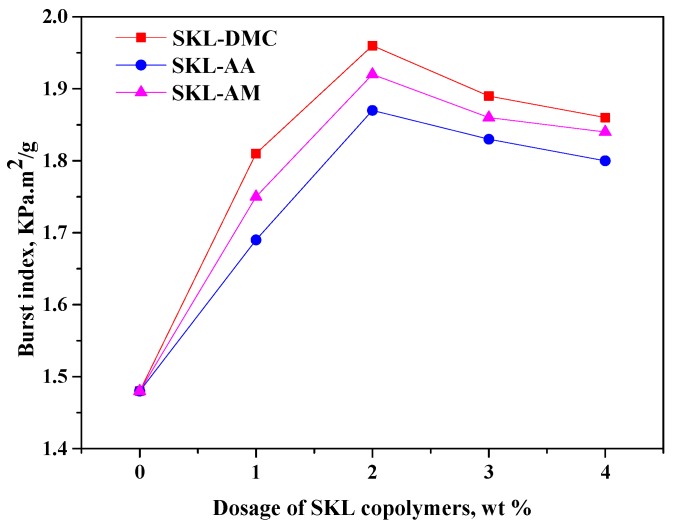
Burst index of paper sheets with various amounts of three additives added (SKL-DMC, SKL-AA, and SKL-AM).

The results in Figure 5, Figure 6 and Figure 7 indicate that all three kinds of SKL copolymers can act as strength additives to improve the tensile, tear, and burst indices of paper sheets. However, the addition of pure kraft lignin as strength additive cannot improve the physical strength properties, even at a 4 wt % dosage (not present in Figure 5, Figure 6 and Figure 7), which is due to the insolubility of kraft lignin in neutral or weak acidic conditions on which the paper sheets are formed. From Figure 5, Figure 6 and Figure 7, it can be observed that the tensile, tear, and burst indices of paper sheets to which the SKL-DMC copolymer was added were the highest among the three additives at the same additive amount, which is due to the high positive charge density that can bind the negatively-charged fibers [34]. The tensile, tear, and burst indices of paper sheets to which the SKL-AA copolymer was added were slightly lower than those of paper sheets to which the SKL-AM copolymer was added. The increase in the strength of the papers was mainly attributed to the formation of H bonding between the SKL copolymers and cellulose fibers or between fibers [18]. When the dosage of SKL copolymers increased from 0% to 1.0%, the tensile, tear, and burst indices increased significantly. At a 2 wt % dosage of SKL copolymers, the value of the tensile, tear, and burst indices reached the maximum values. However, these three strength properties decreased slightly with a further increase in the dosage under the experimental conditions. This was attributed to the presence of an excessive amount of SKL copolymers, which resulted in a lower retention of the SKL copolymers on the fibers. This occurred because the total amount of fibers was constant, and the capacity of fixation by positive/negative ion adsorption was limited by the specific surface of the fibers [35]. Among the prepared three copolymers, the SKL-DMC gave a better performance than SKL-AA or SKL-AM. At the same dosage, SKL-DMC increased the physical strength of paper the most, and SKL-AA increased the physical strength of paper the least.

Table 3 lists the internal bonding strength and brightness of papers in which SKL and SKL copolymers were used as a strength additive at a 2 wt % dosage.

The results show that the internal binding strengths of the SKL-DMC, SKL-AA, and SKL-AM copolymers increased by 61.08%, 49.19%, and 55.14%, respectively, indicating that the increase in the physical strength of papers was mainly attributed to the formation of more H bonding between the cellulose fibers after using lignin copolymers as additives. The brightness decreased slightly, which has little effect on corrugated and cardboard paper.

## 4. Conclusions

In this study, monomers of DMC, AA, and AM were separately grafted onto softwood kraft lignin to prepare three different SKL copolymers. FTIR, ^1^H-NMR, charge density, elemental, and molecular weight analyses confirmed that the monomers were successfully grafted onto SKL. The grafting rates of the SKL-DMC, SKL-AA, and SKL-AM copolymers were 80.35%, 82.70%, and 79.48%, respectively. The molecular weights of the SKL-DMC, SKL-AA, and SKL-AM copolymers reached 4.965 × 10^5^ g/mol, 3.875 × 10^5^ g/mol, and 3.642 × 10^5^ g/mol, respectively, from 2.6 × 10^4^ g/mol of softwood kraft lignin. The application of SKL copolymers as a paper additive for enhancing paper physical properties was studied. The results indicated that at around 2 wt % dosage, the maximum increase in physical strength of paper was achieved. The internal bonding strengths of the SKL-DMC, SKL-AA, and SKL-AM copolymers increased by 61.08%, 49.19%, and 55.14%. Among the prepared three copolymers, SKL-DMC gives a better performance than SKL-AA and SKL-AM.

## Figures and Tables

**Table 1:** Chemical component analysis and properties of poplar APMP pulp.

Ash content (%)	Cellulose content (%)	Pentosan content (%)	Klason lignincontent (%)	Acid dissolved lignin (%)	1% NaOH extract content (%)	Alcohol–benzene extract content (%)	Beating degree (oSR)
0.43	50.38	15.27	17.35	3.56	11.47	1.28	32.5

**Table 2:** Properties of SKL and SKL copolymers.

Samples	SKL	SKL-DMC	SKL-AA	SKL-AM
C (wt %)	63.51	51.53	58.56	55.11
H (wt %)	6.21	6.66	5.62	6.37
O (wt %)	30.57	23.97	31.69	24.90
N (wt %)	0.004	3.003	0.001	8.732
Charge density (mmol/g)	0	+1.425	−6.287	−3.656
Graft ratio (%)	--	80.35	82.70	79.48
Molecular formula	C_9_H_10.56_O_3.25_	C_9_H_13.96_O_3.14_N_0.45_	C_9_H_10.37_O_3.85_	C_9_H_12.48_O_3.05_N_1.22_
*Mn* (g/mol)	1.725 × 10^4^	4.352 × 10^5^	3.267 × 10^5^	2.145 × 10^5^
*Mw* (g/mol)	2.600 × 10^4^	4.965 × 10^5^	3.875 × 10^5^	3.642 × 10^5^
*Mw/Mn*	1.51	1.14	1.19	1.70

**Table 3:** Internal bonding strength and brightness of papers.

Samples	SKL	SKL-DMC	SKL-AA	SKL-AM
Internal bonding strength (J/m^2^)	185	298	276	287
Brightness (%ISO)	75.6	74.8	75.2	75.0
